# A multifactorial approach to tacrolimus therapeutic drug monitoring in complex liver transplantation: a case report

**DOI:** 10.3389/fmed.2026.1837572

**Published:** 2026-07-24

**Authors:** Limian Liang, Tian He, Yiming Huang, Min Wu, Jiajia Liu, Shiyun Chen, Wei Li, Miaona Liu, Pusen Wang

**Affiliations:** 1 Department of Pharmacy, Shenzhen Third People’s Hospital, Shenzhen, Guangdong, China; 2 Department of Liver Surgery and Organ Transplantation Center, Shenzhen Third People’s Hospital, Shenzhen, Guangdong, China

**Keywords:** complex cases, dose adjustment, hematocrit, liver transplantation, pharmacokinetics, tacrolimus, therapeutic drug monitoring

## Abstract

This case describes a structured, multifactorial framework for interpreting tacrolimus therapeutic drug monitoring in a complex liver transplant recipient, with hypothesis-generating implications for clinical decision-making. This was a single-patient case report with serial therapeutic drug monitoring and longitudinal clinical follow-up. The clinical course of a 33-year-old male liver transplant recipient was documented in detail. During a 90-day hospitalization, the patient underwent two liver transplantations and experienced abnormal liver function, progressive anemia, and severe diarrhea, accompanied by marked fluctuations in tacrolimus trough concentrations (C_tac_). Hematocrit (HCT)-corrected standardized concentrations (C_std_) were calculated using the formula C_std_ = Cmeasured × (45/HCT[%]) as an interpretive aid for whole-blood C_tac_ during marked anemia. Across serial measurements in this single patient, tacrolimus whole-blood trough concentrations showed a positive descriptive association with HCT. Three distinct pharmacokinetic phases were identified: (1) early severe hepatic dysfunction, during which the expected CYP3A5 extensive-metabolizer phenotype appeared to be masked (peak C/D ratio: 7.70), likely attributable to compromised hepatic metabolic capacity; (2) recovery with markedly reduced HCT associated with apparently low whole-blood trough concentrations (nadir measured C: 3.2 ng/mL; corrected C_std_: 7.0 ng/mL) supporting cautious interpretation during severe anemia;and (3) inflammatory diarrhea associated with increased tacrolimus exposure (C/D ratio increased approximately 55%), possibly related to altered intestinal drug transporter and metabolic enzyme activity. Dose adjustments guided by the structured strategy successfully maintained drug exposure within the target range throughout the 90-day course without confirmed rejection episodes or severe tacrolimus-related toxicity. This case illustrates how a structured, multifactorial approach to tacrolimus therapeutic drug monitoring interpretation may assist clinical decision-making in complex post-liver-transplant settings. The proposed framework integrates pharmacological mechanisms with clinical evidence to address challenges in tacrolimus TDM interpretation. Prospective validation is required before the proposed framework can be recommended as a generalizable clinical strategy.

## 1 Introduction

Patients undergoing liver transplantation require lifelong administration of potent immunosuppressive agents to prevent graft rejection (1). Tacrolimus is widely used as a cornerstone immunosuppressant (2). As a macrolide immunosuppressive agent, tacrolimus inhibits calcineurin activity and blocks T-lymphocyte activation. The drug has a narrow therapeutic window and exhibits substantial interindividual pharmacokinetic variability (3, 4). Minor fluctuations in blood concentrations may result in insufficient efficacy or drug-related toxicity. Previous case reports have predominantly examined the impact of single factors on tacrolimus exposure, whereas studies addressing the interaction of multiple factors remain limited. Here, we report a liver transplant recipient who sequentially developed hepatic artery thrombosis, progressive anemia, septic shock necessitating re-transplantation, and severe inflammatory diarrhea—all within a 90-day hospitalization—resulting in overlapping effects on tacrolimus pharmacokinetics. This case aims to illustrate how individualized dosing can be achieved through integration of clinical evidence and therapeutic drug monitoring (TDM) data, and to propose a structured dose adjustment strategy for clinical practice and education.

## 2 Case presentation

### 2.1 Pre-transplant clinical course

A 33-year-old Chinese man was admitted on June 2, 2025, with hepatitis B virus–related acute-on-chronic liver failure (HBV-ACLF), hepatic encephalopathy, and intra-abdominal infection. Laboratory tests showed total bilirubin of 425 μmol/L, ALT 1099 U/L, AST 1086 U/L, INR 2.74, and a MELD score of 31. Given the critical condition, he underwent living-donor left-lobe liver transplantation on June 9, 2025, with his sister as the donor. Basiliximab was administered as induction immunosuppressive therapy. Preoperative evaluation revealed the recipient genotypes CYP3A4 *1/*1G, CYP3A5 *1/*3, and ABCB1 AA, while the donor genotypes were CYP3A4 *1G/*1G and CYP3A5 *1/*3. The patient and his caregivers received standardized medication education, and no missed doses, incorrect doses, or unauthorized discontinuation of medication occurred during hospitalization.

### 2.2 Postoperative phase 1: severe hepatic dysfunction and progressive anemia (POD 1–59)

Early after transplantation, hepatic artery thrombosis occurred, requiring emergency angioplasty and stent implantation on postoperative day (POD) 3. The patient subsequently exhibited hepatic dysfunction (total bilirubin 254–288 μmol/L) and worsening intra-abdominal infection, which improved after antibiotic and supportive therapy, allowing transfer to the general ward. During this period, progressive anemia (Hemoglobin 87 g/L) developed, with hematocrit (HCT) decreasing to 26.8%. Due to severe perioperative infection, immunosuppressive therapy was delayed until POD 8.

The postoperative immunosuppressive regimen consisted of oral tacrolimus (Prograf®, Astellas Pharma Co., Ltd.) 2 mg every 12 h, with dose adjustment based on the infection status and target trough concentrations (5–7 ng/mL); enteric-coated mycophenolate sodium (Myfortic®, Novartis Pharma GmbH) 540 mg every 12 h, adjusted according to target concentrations of 6–10 ng/mL during the first month and 4–8 ng/mL thereafter (5); and methylprednisolone (Medrol®, Pfizer Italia s.r.l.), administered intravenously and gradually tapered to oral dosing at 4 mg once daily. No concomitant medications with significant effects on *CYP3A4* or *CYP3A5* activity were used.

On POD 11, C_tac_ increased unexpectedly (Table 1) to 15.4 ng/mL, with a markedly elevated concentration-to-dose (C/D) ratio of 5.13, which was inconsistent with genotype-based predictions. The tacrolimus dose was reduced to 1.5 mg every 12 h; yet, trough concentrations remained elevated (11.3–18.7 ng/mL). Until POD 18, tacrolimus was discontinued for 2 days and subsequently resumed at a reduced dose of 0.5 mg every 12 h, During this period, the patient’s infection was controlled, and trough concentrations returned to the target range (6.4–7.7 ng/mL). From POD 22 onward, the patient developed intra-abdominal infection with gradual deterioration and abnormal elevation of total bilirubin levels. A liver biopsy was performed, and the pathological findings suggested infection-related liver injury. On POD 51, the patient was transferred to the intensive care unit due to septic shock with carbapenem-resistant *Klebsiella pneumoniae* and *Enterococcus faecium* infections. Immunosuppressive agents were discontinued, artificial liver support was initiated, and a second transplantation was planned.

**Table 1:** Tacrolimus dosing, key clinical parameters, and HCT-corrected standardized concentrations during the postoperative course.

POD	Tacrolimus daily dose (mg)	Daily FK506 dose (mg)	FK506 trough concentration* (ng/mL)	C_tac_ (ng/mL)	C/D ratio (ng/mL per mg)	C_std_ (ng/mL) [HCT-corrected]	Total Bilirubin (μmol/L)	AST (U/L)	ALT (U/L)	HCT (%)	Albumin (g/L)	Diarrhea episodes (per day)
8	4	4	0	—	0.00	—	201.4	182	274	26.8	33.0	—
10	4	4	8.1	**8.1**	2.03	12.7*	198.6	105	137	28.6	37.5	—
11	3	3	15.4	**15.4**	5.13	24.2*	250.0	123	159	28.6	38.1	—
12	3	3	11.3	**11.3**	3.77	17.5*	195.0	59	78	29.1	37.3	—
14	3	3	18.7	**18.7**	6.23	28.7*	130.0	63	60	29.3	27.3	—
22	1	1	7.7	7.7	7.70	15.5*	270.4	84	86	22.4	37.6	—
23	1	1	6.4	6.4	6.40	12.6*	269.6	57	87	22.8	35.8	—
29	1	1	6.6	6.6	6.60	14.8*	254.7	197	210	20.1	31.5	—
39	1	1	5.4	5.4	5.40	10.4*	199.7	106	132	23.4	33.1	—
42	1	1	5.1	5.1	5.10	12.0*	277.6	152	151	19.2	31.8	—
49	1	1	5.3	5.3	5.30	14.5*	287.9	110.4	140.4	16.4	30.0	—
66	4	4	4	4.0	1.00	9.0*	67.6	33.7	80.0	20.0	32.0	—
67	4	4	3.2	3.2	0.80	7.0*	61.4	33.2	68.9	20.6	31.9	—
70	4	4	5.9	5.9	1.48	14.0*	44.7	25.0	34.0	18.9	40.6	—
73	4	4	8	**8.0**	2.00	18.1*	46.1	33.0	22.0	19.9	37.9	7
79	3	3	6.4	6.4	2.13	13.3*	67.2	44.0	35.0	21.7	32.9	3
81	3	3	4.9	4.9	1.63	10.7*	40.1	31.0	27.0	20.6	30.8	4
84	3	3	5.4	5.4	1.80	11.3*	22.5	27.8	16.3	21.5	29.3	3
87	3	3	5.3	5.3	1.77	9.7*	24.2	24.8	14.0	24.7	38.3	2
90	3	3	5.5	5.5	1.83	10.4*	20.5	46.8	11.3	23.9	36.1	3

Row shading: Blue = Phase 1 (POD 1–59, severe hepatic dysfunction and anemia); Green = Phase 2 (POD 60–79, second liver transplantation and acute diarrhea); Amber = Phase 3 (POD 80–90, metabolic stabilization and chronic diarrhea). Italic C_std_ values: HCT < 30%, where correction is clinically indicated [threshold per Brunet et al. (4) Second Consensus Report]. Bold C_tac_ values: supratherapeutic concentration (> 7 ng/mL) at time of TDM-guided dose reduction. C_std_ values were interpreted in conjunction with the same reference band used for visual comparison in Figures 2, 3; this band was intended to facilitate interpretation across phases rather than define a fixed therapeutic target for all timepoints. * According to the liver transplantation protocol established at our center with reference to guideline recommendations (5), the initial target C_tac_ after transplantation was set at 5–7 ng/mL for low-immunological-risk recipients receiving basiliximab induction therapy. If postoperative infection occurred, the target concentration was reduced or tacrolimus therapy was temporarily suspended as appropriate. During the entire 90-day treatment period, the patient did not receive any medications known to significantly interfere with hepatic CYP3A4 or CYP3A5 activity, as listed in the tacrolimus prescribing information, including triazole antifungal agents, macrolide antibiotics, calcium channel blockers, protease inhibitors, rifamycins, antiepileptic drugs, or herbal preparations. POD, postoperative day; C_tac_, tacrolimus trough whole-blood concentration measured by EMIT (LLOQ2.0 ng/mL, intra-assay CV < 9.6%); C/D ratio, the blood concentration normalized by the dose; C_std_, HCT-corrected standardized tacrolimus concentration calculated; TBil, total bilirubin; AST, aspartate aminotransferase; ALT, alanine aminotransferase; HCT, hematocrit; Alb, albumin.

### 2.3 Postoperative phase 2: second liver transplantation and acute diarrhea (POD 60–79)

On POD 60, the patient underwent a second liver transplantation using a whole liver graft from a deceased donor. Basiliximab was administered as induction immunosuppressive therapy. Preoperative evaluation showed the donor genotypes were CYP3A4 *1/*1G and CYP3A5 *1/*3. After the second transplantation, liver function improved markedly, with total bilirubin levels ranging from 45 to 68 μmol/L, and infectious symptoms were effectively controlled. On POD 64, maintenance immunosuppressive therapy was reinitiated. Considering the improvement in liver function and infection status, the initial tacrolimus dose was set at 2 mg every 12 h, with a target trough concentration of 5–7 ng/mL. Unexpectedly, despite a fourfold increase in dose compared with the previous stage, C_tac_ reached only 3.2–5.9 ng/mL, which was associated with markedly reduced HCT levels (18.9–20.0%) during this period.

From POD 71 onward, the patient developed severe watery diarrhea (6–7 episodes per day), accompanied by low-grade fever and elevated C-reactive protein levels. Although the tacrolimus dose remained unchanged, trough concentrations increased to 8.0 ng/mL, indicating altered tacrolimus disposition during acute diarrhea, Accordingly, on POD 79, the tacrolimus dose was reduced to 1.5 mg every 12 h, and therapeutic drug monitoring was intensified.

### 2.4 Postoperative phase 3: metabolic stabilization and chronic diarrhea (POD 80–90)

During this phase, liver function approached near-normal levels, with total bilirubin ranging from 21 to 67 μmol/L, while HCT remained low (20.6–24.7%). The patient experienced persistent diarrhea (3–4 episodes per day). Despite ongoing diarrhea, the C/D ratio increased to 1.6–2.1, suggesting that chronic intestinal inflammation may have continuously downregulated P-glycoprotein (P-gp) and CYP3A expression, increasing tacrolimus bioavailability. The tacrolimus dose was maintained at 1.5 mg every 12 h, achieving stable therapeutic trough concentrations of 5.3–6.4 ng/mL without clinically confirmed rejection or nephrotoxicity.

## 3 Analytical methods

Whole-blood C_tac_ were measured using a validated enzyme multiplied immunoassay technique (EMIT) on the Siemens Viva-ProE system (Syva® EMIT® 2000 Tacrolimus Assay), with a lower limit of quantification (LLOQ) of 2.0 ng/mL and intra-assay coefficient of variation (CV) < 9.6%. Samples were collected 12 h (±30 min) after the evening dose under fasting conditions, processed within 2 h of collection, and analyzed in duplicate. Dose changes were avoided before blood sampling. During Phase 1, more frequent therapeutic drug monitoring was performed due to the patient’s clinical condition. In Phases 2 and 3, the interval between dose adjustment and blood concentration measurement was 3 days or longer. Hematological parameters (HCT, hemoglobin, and red blood cell count) were measured using a calibrated automated hematology analyzer. Donor and recipient pharmacogenetic genotyping for CYP3A4 (*1, *1G), CYP3A5 (*1, *3), and ABCB1 (AA, AG, GG) was performed using fluorescence hybridization sequencing.

## 4 Data processing and analysis

During POD 1–90, C_tac_ (C0), HCT, liver function parameters, and recorded diarrhea episodes were prospectively collected at each TDM timepoint. HCT-corrected standardized concentrations (C_std_) were calculated for all observations using the formula C_std_ = C_measured_ × (45/HCT[%]), which standardizes measured whole-blood concentrations to a reference HCT of 45% (adult normal value). These corrected values were used as an interpretive aid for whole-blood C_tac_ during marked anemia and should not be considered a direct surrogate for unbound tacrolimus exposure (6). This correction was applied to support interpretation when HCT deviates by > 15% from baseline or falls below 30%. Pearson correlation analysis and linear regression were performed to evaluate the relationship between HCT and measured C_tac_ across all 20 TDM observations with concurrent HCT data. The C/D ratio was calculated by dividing the C0 concentration of the drug by the daily dose. Statistical analyses were performed using SPSS v28.0 and R v4.5.1. Given the single-patient observational design, all statistical outputs are presented descriptively; longitudinal autocorrelation was noted as a methodological limitation.

## 5 Results

### 5.1 Impact of liver function on tacrolimus concentration

**Figure 1:**
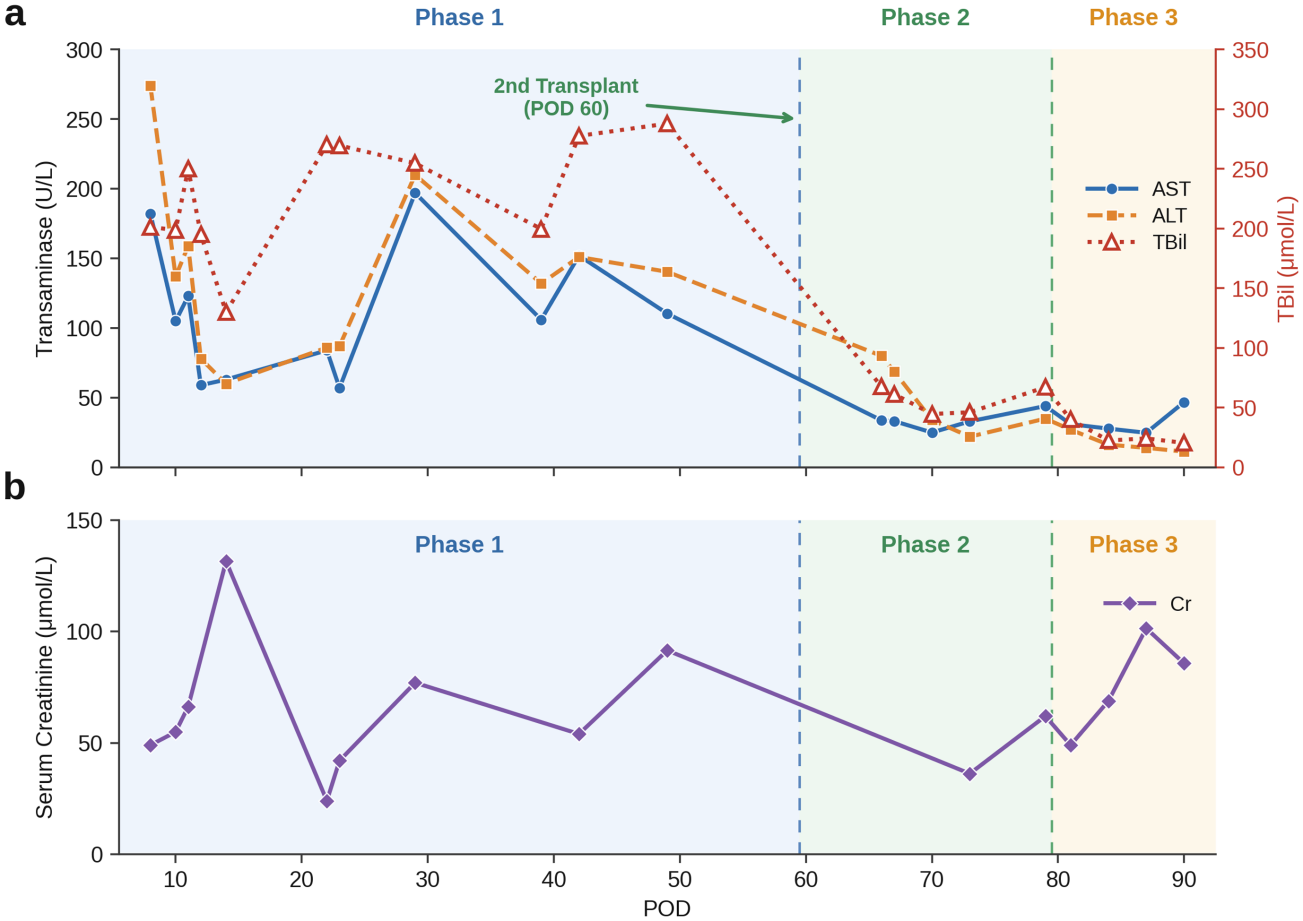
Evolution of hepatic and renal function across the three post-transplant clinical phases. **(a)** Temporal changes in aspartate aminotransferase (AST), alanine aminotransferase (ALT), and total bilirubin (TBil). **(b)** Temporal changes in serum creatinine (Cr). The vertical dashed lines separate the three clinical phases, and the second transplantation on postoperative day 60 is indicated in panel (a). POD, postoperative day.

As illustrated in Figure 1, three distinct pharmacokinetic phases were identified. During Phase 1 (POD 1–59), severe hepatic dysfunction, progressive anemia, and markedly reduced tacrolimus clearance were observed, with a peak C/D ratio of 7.70 on POD 22. During Phase 2 (POD 60–79), recovery of hepatic function after the second transplantation was accompanied by near-normalization of tacrolimus metabolism (peak C/D ratio 2.13); paradoxically low measured concentrations during this phase were attributable predominantly to profound anemia (see Section 5.2). During Phase 3 (POD 80–90), hepatic function approached normal levels and tacrolimus metabolism remained stable (C/D ratio 1.63–1.83). The temporal evolution of total bilirubin, AST, ALT, and serum creatinine is depicted in Figure 1.

### 5.2 Impact of HCT on tacrolimus concentration

**Figure 2:**
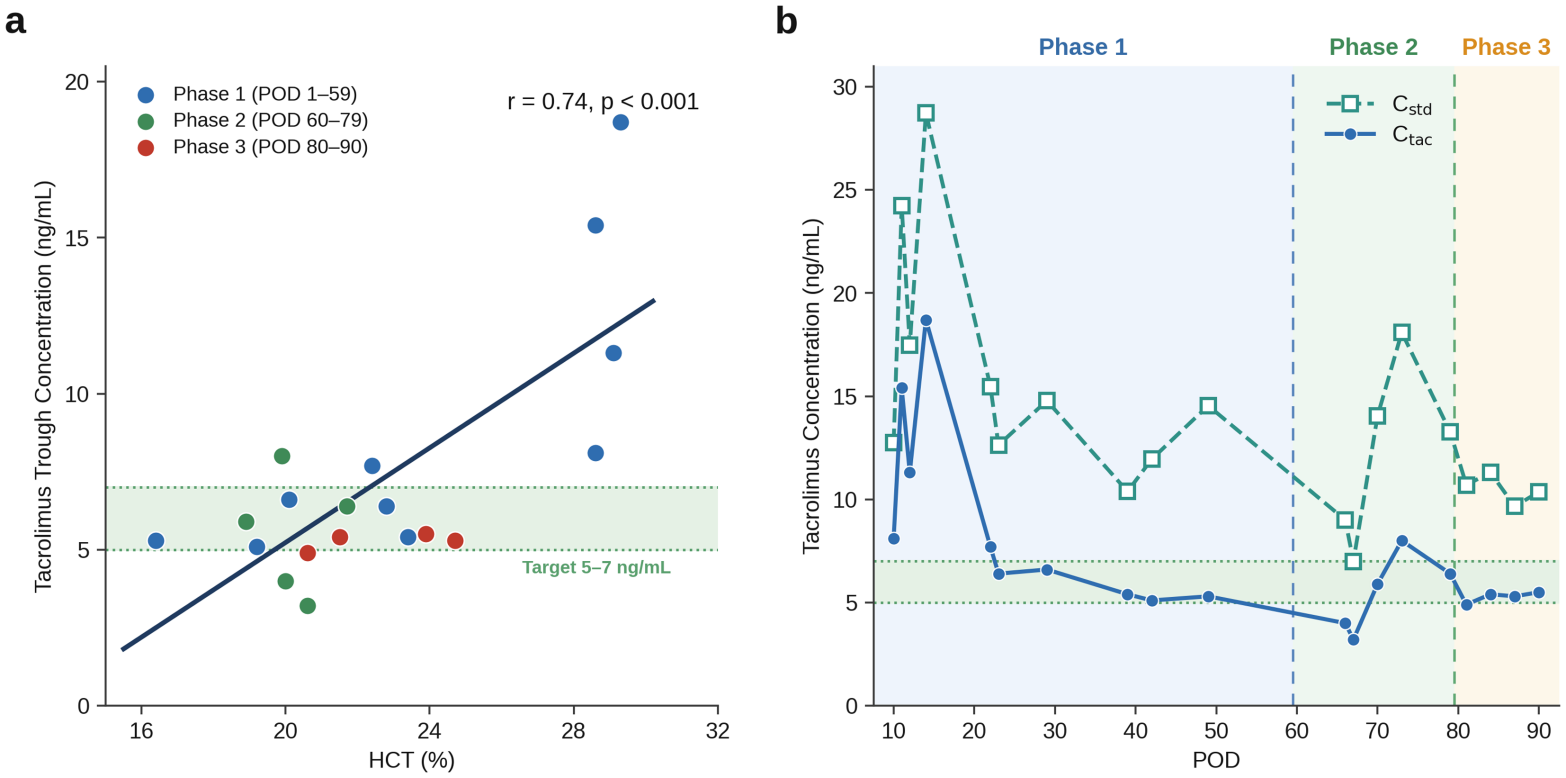
**(a)** Descriptive association between hematocrit (HCT) and measured tacrolimus whole-blood trough concentration (Ctac). **(b)**. Measured tacrolimus whole-blood trough concentration and HCT-corrected standardized concentration (Cstd) over the postoperative course. The shaded band represents the tacrolimus reference range used for visual comparison and should not be interpreted as a fixed therapeutic target for every postoperative stage. HCT, hematocrit; Ctac, tacrolimus whole-blood trough concentration; Cstd, HCT-corrected standardized concentration; POD, postoperative day.

C_tac_ showed a positive descriptive association with HCT across all available TDM timepoints (Pearson r = 0.74, 95% CI: 0.44–0.89, *p* < 0.001). However, this association should be interpreted cautiously because the data consist of repeated longitudinal measurements from a single patient and are confounded by concurrent changes in liver function, dose adjustments, diarrhea, infection, albumin, and retransplantation. During the anemia phase (HCT nadir 16.4%, POD 49), the measured trough concentration was as low as 3.2 ng/mL (POD 67), without HCT contextualization, could be misinterpreted as subtherapeutic whole-blood exposure. Applying the standardized correction formula C_std_ = Cmeasured × (45/HCT[%]), yielded the corrected concentration at POD 67 of 7.0 ng/mL—within the therapeutic window. As shown in Figure 2, C_std_ values were less variable than measured whole-blood trough concentrations across Phases 1–3 and remained within or above the prespecified reference range used for clinical interpretation, while measured whole-blood concentrations showed greater variability attributable to HCT fluctuations. This discordance between measured and corrected concentrations highlights the potential value of HCT contextualization in anemic transplant recipients. Dose escalation based solely on measured whole-blood concentrations during marked anemia may increase the risk of overexposure and should be interpreted cautiously.

### 5.3 Impact of gastrointestinal barrier function on tacrolimus concentration

**Figure 3:**
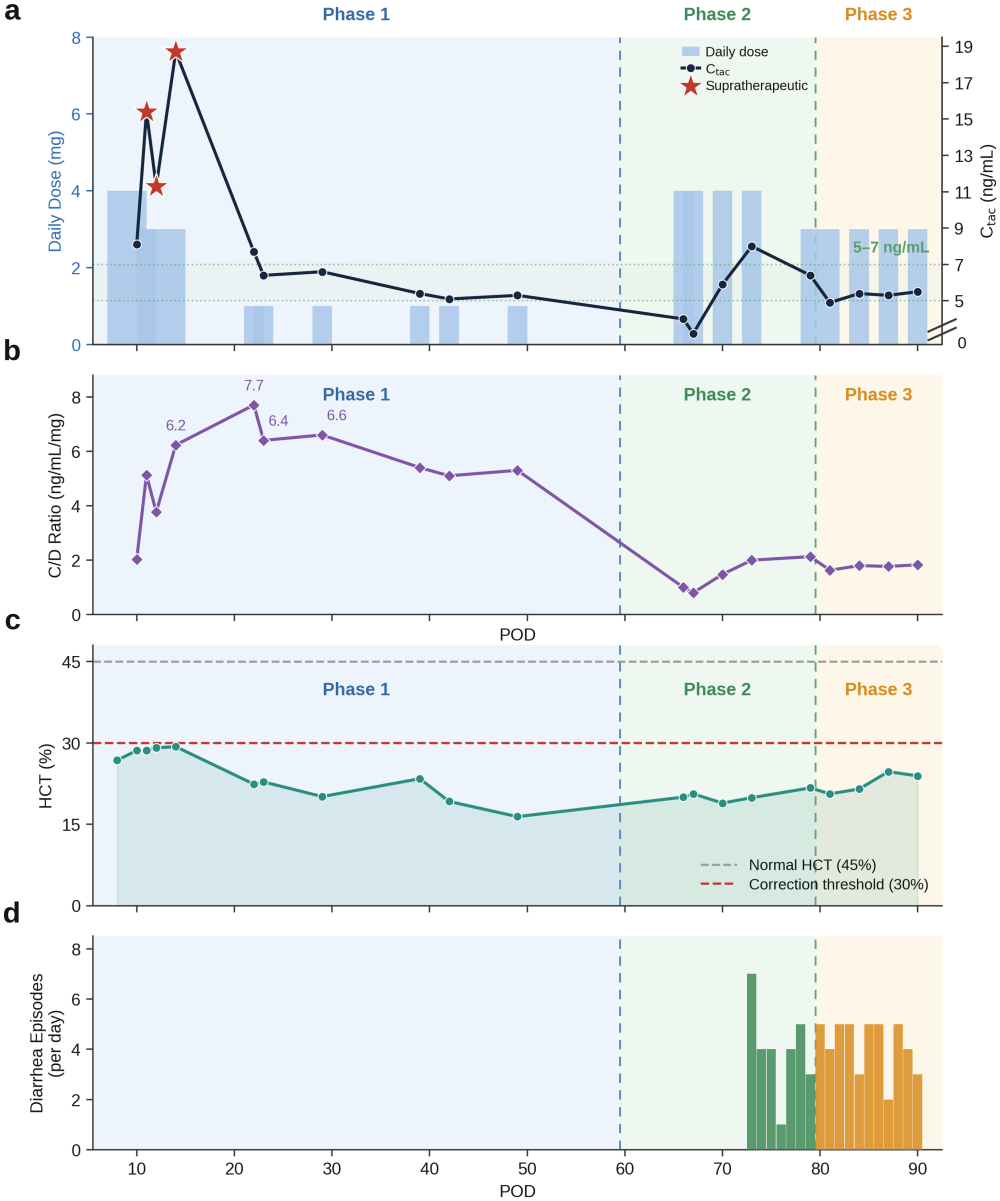
Comprehensive multi-panel visualization of tacrolimus pharmacokinetics and clinical covariates over the 90-day postoperative course. **(a)** Daily tacrolimus dose and measured whole-blood trough concentration (Ctac), with concentrations above the reference range indicated by red stars. **(b)** Tacrolimus concentration-to-dose (C/D) ratio. **(c)** Hematocrit (HCT). **(d)** Recorded diarrhea frequency, expressed as episodes per day. The vertical dashed lines separate the three clinical phases. The shaded tacrolimus band represents the reference range used for visual comparison across phases and should not be interpreted as a fixed therapeutic target applied uniformly throughout the clinical course. C/D ratio, concentration-to-dose ratio; Ctac, tacrolimus whole-blood trough concentration; HCT, hematocrit; POD, postoperative day.

As shown in Figure 3, during the diarrhea episode, the C/D ratio increased by approximately 55% compared with baseline. Through serial dose adjustment and intensified therapeutic drug monitoring, tacrolimus whole-blood trough concentrations were maintained within the prespecified reference range used in clinical management.

### 5.4 Multifactor-based structured dose adjustment strategy

To address the multifactorial pathophysiological changes in complex post-liver transplantation cases, a multifactoria structured tacrolimus dose adjustment framework is proposed (Supplementary Figure S1). This framework integrates pharmacogenomics, hepatic function assessment, C_std_ gastrointestinal barrier evaluation, drug–food interaction screening, and closed-loop monitoring—moving beyond conventional single-threshold, concentration-driven dose adjustment. During the 90-day treatment period, the patient did not experience biopsy-proven rejection confirmed by liver histopathological examination, nor were any severe tacrolimus-related toxicities observed, such as neurotoxicity or renal dysfunction.

#### 5.4.1 Genotype-based initial dosing strategy

Pharmacogenetic polymorphisms constitute a fundamental determinant of tacrolimus pharmacokinetics. Tacrolimus is primarily metabolized by hepatic *CYP3A4* and *CYP3A5* enzymes, and genetic polymorphisms in these genes can result in substantial interindividual variability in enzyme activity (7). In addition, the *ABCB1* gene encodes P-gp, which participates in tacrolimus pharmacokinetics by regulating intestinal absorption and drug distribution.

*Decision recommendations*:

*CYP3A5*: Pre-transplant genotyping of CYP3A5 is recommended for both donors and recipients. CYP3A5 expression is generally associated with lower dose-adjusted C_tac_ and higher dose requirements. In liver transplantation, however, the relative contributions of recipient intestinal and donor hepatic CYP3A5 may shift over time; therefore, genotype should inform, but not solely determine, initial dosing and subsequent interpretation Therapeutic drug monitoring should be used to guide subsequent dose adjustments (8).

*CYP3A4*: Existing studies show heterogeneous findings (9–11). Therefore, in clinical practice, individualized dose adjustment should be performed in combination with therapeutic drug monitoring (TDM). The impact of this polymorphism appears to be limited mainly to patients with the CYP3A5 *3/3 slow-metabolizer phenotype; thus, it is not recommended to use *CYP3A4* polymorphism as an independent basis for dosing decisions. Only when a patient carries the CYP3A5 *3/3 genotype and repeatedly exhibits excessively high C_tac_ should dose adjustment with reference to the *CYP3A4 1G* polymorphism be considered (12).

*ABCB1*: Variants in the ABCB1 gene may affect P-gp –mediated intestinal absorption efficiency; yet, the association between its polymorphisms and P-gp function shows considerable interindividual variability and requires individualized adjustment based on TDM. ABCB1 polymorphisms may contribute to interindividual variability in tacrolimus absorption, but current evidence in liver transplantation remains inconsistent. Therefore, ABCB1 was considered a supportive rather than stand-alone factor in dose interpretation.

#### 5.4.2 Dynamic assessment of hepatic function

The liver is the primary organ responsible for tacrolimus metabolism, and the degree of hepatic functional recovery after liver transplantation directly affects drug clearance. Hepatocellular injury can downregulate *CYP3A* enzyme activity, while post-transplant fluctuations in liver function are commonly associated with ischemia–reperfusion injury, rejection episodes, or infection.

*Decision recommendations*: Hepatic function indicators should be evaluated using total bilirubin (TBIL), transaminases, and MELD score. After hepatic metabolism, approximately 95% of tacrolimus metabolites are excreted via bile;thus, biliary obstruction can markedly increase blood tacrolimus concentrations (13). In addition, studies have shown reduced *CYP3A* protein expression in cholestatic liver disease, which may further decrease drug clearance (14).

During the early post-transplant period or in cases of delayed graft function (DGF), if hyperbilirubinemia or increasing MELD scores are present, loading doses should be avoided. A “low-dose initiation with daily upward titration” strategy is recommended, together with increased TDM frequency.

#### 5.4.3 HCT-adjusted evaluation

Tacrolimus exhibits a strong affinity for erythrocytes (15), and whole-blood trough concentrations are highly dependent on HCT, whereas its pharmacological effects are mainly driven by the unbound (free) fraction. When HCT is markedly reduced, fewer erythrocyte binding sites are available, leading to a pseudo-decrease in measured whole-blood concentrations, while free drug concentrations may remain unchanged or even increase. Blind dose escalation under these circumstances may increase the risk of adverse events.

*Decision recommendations*: In patients with anemia or complex clinical conditions, HCT and other covariates should be integrated to optimize TDM interpretation (4). C_std_ should be calculated using HCT-correction formulas and used to guide dose adjustment. When HCT deviates from baseline by >15% (or HCT < 30%), C_std_ should be calculated and dosing decisions based on the corrected value to better reflect true drug exposure.

#### 5.4.4 Assessment of gastrointestinal barrier function

The impact of diarrhea on tacrolimus pharmacokinetics is bidirectional; yet, inflammatory diarrhea typically leads to abnormally increased blood concentrations. This may result from changes in drug solubility, altered intestinal permeability affecting absorption, and reduced activity of *CYP3A4* and/or P-gp in enterocytes (16–18).

*Decision recommendations*:

- Mild diarrhea: Maintain the current dose with close monitoring.
- Moderate to severe diarrhea: Due to the risk of increased absorption, a prophylactic tacrolimus dose reduction of approximately 30% is recommended (16). If diarrhea causes highly unstable absorption, temporary conversion to intravenous administration may be considered.

#### 5.4.5 Evaluation of drug–food interactions

As a substrate of *CYP3A4/5* and P-gp, tacrolimus is highly susceptible to the influence of concomitant medications and foods (19, 20). After liver transplantation—particularly in complex cases requiring multidrug therapy—drug interactions represent a major external cause of pronounced fluctuations in tacrolimus blood concentrations.

*Decision recommendations*:

- Drug–drug interactions: Clinicians may utilize computerized prescription review systems and consult drug labels, the Lexidrug database, and clinical literature to stratify concomitant medications into three risk categories based on their effects on *CYP3A4/5* and P-gp.

1. Avoid combination: e.g., protease inhibitors (21, 22); such combinations should be strictly avoided.
2. Consider therapy modification: rifamycins and azole antifungals; tacrolimus dose adjustment is recommended on the day of co-administration with increased monitoring frequency (23).
3. Monitor therapy: Calcium channel blockers such as nifedipine should be administered with dose adjustments guided by tacrolimus concentration (24).

- Drug–food interactions: Certain dietary components can markedly interfere with tacrolimus metabolism; thus, food effects should not be overlooked when unexplained concentration variability occurs (25, 26). Foods rich in furanocoumarins inhibit intestinal *CYP3A4* activity (27, 28), resulting in significantly increased tacrolimus exposure and should be clearly listed as lifelong contraindications in post-transplant education. St. John’s Wort is a potent *CYP3A4* inducer (29) and can cause abrupt decreases in tacrolimus concentrations (30). Food intake—especially high-fat meals—delays gastric emptying and reduces the absorption rate of tacrolimus (31). Alcohol consumption may further burden hepatic function, interfere with drug metabolism, and increase the risk of gastrointestinal mucosal injury.

#### 5.4.6 Other physiological factors

In addition to the factors described above, several other factors may potentially interfere with tacrolimus blood concentrations. For example, severe hypoalbuminemia may increase the proportion of free tacrolimus and thereby increase the risk of drug toxicity; however, current evidence remains controversial (32–34). The effects of critical pathophysiological conditions on drug metabolism are systemic. Infection and systemic inflammatory response syndrome may continuously suppress hepatic and intestinal CYP3A4/5 enzyme activity, significantly reducing metabolic clearance and resulting in drug accumulation (35, 36). Blood transfusion may increase red blood cell counts, thereby affecting tacrolimus distribution and leading to elevated whole-blood tacrolimus concentrations (37, 38).

Decision recommendation: High-quality evidence systematically confirming the effects of the above factors is still lacking. Therefore, in complex cases involving these abnormal physiological conditions, their potential impact on tacrolimus blood concentrations should be carefully evaluated.

#### 5.4.7 Analytical methods and timing

In addition, variability related to analytical methodology should also be taken into consideration. Although enzyme multiplied immunoassay technique (EMIT) can satisfy the requirements of routine monitoring, its accuracy may decrease in the presence of marked fluctuations in endogenous substances or cross-reactivity with metabolites (39).

Decision recommendation: When tacrolimus blood concentrations are clearly inconsistent with the clinical condition, the limitations of the analytical method should be comprehensively evaluated. If necessary, liquid chromatography–tandem mass spectrometry (LC–MS/MS), which has higher specificity, should be used for confirmation to avoid inappropriate dose adjustment and adverse consequences caused by analytical bias.

#### 5.4.8 Closed-loop monitoring

A collaborative monitoring system involving physicians, clinical pharmacists, nurses, patients, and caregivers should be established, using “events” rather than time alone as triggers.

- Assess: Regularly evaluate medication adherence, review clinical data, and analyze sources of pharmacokinetic variability in conjunction with genotype background.
- Adjust: When risk events occur, initiate intensified monitoring and adjust dosing until parameters return to baseline.
- Verify: Reassess promptly after dose adjustment to confirm that drug exposure has returned to the target range and monitor for related adverse effects, completing the decision-making loop.

## 6 Discussion

This case provides a detailed documentation of the pharmacokinetic evolution of tacrolimus in a patient undergoing two liver transplantations complicated by severe anemia, infection, and diarrhea. Unlike routine cases, this patient exhibited characteristics of “multifactorial overlap with temporal dynamics,” in which single-dimensional assessment was insufficient to meet the requirements of precision therapy. By deconstructing this case, we demonstrate how genetic polymorphisms, HCT levels, and gastrointestinal barrier function dynamically interact and interfere with tacrolimus blood concentrations in complex clinical settings,illustrating the clinical plausibility and potential utility of a multifactorial structured dose adjustment framework.

### 6.1 Genotype-guided initial dose prediction

Pharmacogenomics provides a static reference for the initial dosing of tacrolimus; however, its applicability during the early period after liver transplantation should be evaluated in the context of dynamic changes in graft function. Tacrolimus metabolism is influenced by both recipient intestinal CYP3A5 activity and donor liver CYP3A5 activity, and the relative contributions of these two components may change over time. However, current studies are limited by small sample sizes and inconsistent conclusions (40–42). In this case, the recipient carried the CYP3A5 *1/*3 genotype, suggesting preserved intestinal CYP3A5 expression capable of contributing to the first-pass metabolism of orally administered tacrolimus. Both the initial donor and the retransplantation donor carried the same CYP3A5 *1/*3 genotype; therefore, there was no difference in the hepatic CYP3A5 expression status between the two transplanted grafts. During the early period after the first transplantation, severe graft dysfunction and infection markedly impaired hepatic metabolic capacity, resulting in unexpectedly elevated tacrolimus blood concentrations (with a C/D ratio as high as 7.70), which may have temporarily masked the effect of the rapid-metabolizer genotype. A modeling analysis by Huang et al. (43) on CYP3A5 genotype and time-dependent metabolic patterns in Chinese liver transplant recipients demonstrated that the apparent clearance of tacrolimus increased by 0.57 L/h per day within the first 28 days after transplantation (95% CI: 0.45–0.66), suggesting that early graft functional recovery may influence CYP3A5 expression and thereby affect drug metabolism. Following retransplantation on postoperative day 60, rapid recovery of liver function, resolution of cholestasis, control of sepsis, and gradual correction of anemia collectively promoted the recovery of hepatic CYP3A5 activity to a level consistent with the donor genotype, while the recipient’s intestinal CYP3A5 activity continued to contribute to tacrolimus metabolism. As a result, the patient’s overall metabolic phenotype gradually shifted toward the genotype-predicted state, requiring dose escalation to achieve therapeutic concentrations. It should be emphasized that the masking effect of early hepatic dysfunction on the CYP3A5 metabolic phenotype observed in this case should be regarded as a clinical interpretation, because multiple dynamic factors, including liver function, biliary excretion, gastrointestinal integrity, and hematocrit, jointly influence tacrolimus disposition. Therefore, in complex liver transplant cases, dosing based solely on genotype may be misleading, highlighting the clinical value of a multifactor-based structured dose adjustment strategy.

### 6.2 Impact of HCT fluctuations on tacrolimus concentration

The observed positive correlation between HCT and tacrolimus whole-blood trough concentrations (Pearson r = 0.74, *p* < 0.001) aligns with established pharmacokinetic principles and prior reports (4, 44). Population pharmacokinetic models excluding HCT as a covariate demonstrate poor predictive performance, underscoring its importance in model extrapolation (45).

At the nadir anemia phase (HCT 16.4%, POD 49), measured concentrations dropped to 3.2 ng/mL (POD 67)—a value conventionally prompting dose escalation. C_std_ at this timepoint reached 7.0 ng/mL, remaining within the therapeutic window. This correction averted unnecessary dose increases that could have led to free-drug overexposure, neurotoxicity, or nephrotoxicity. The case thus illustrates clinical application of the Størset et al. HCT-correction framework (6) in a retransplantation context, though generalizability requires prospective validation across diverse clinical settings.

### 6.3 Influence of gastrointestinal barrier function on tacrolimus concentration

The impact of diarrhea on tacrolimus pharmacokinetics is complex. Traditionally, accelerated intestinal motility associated with diarrhea is believed to shorten drug absorption time and reduce blood concentrations. In this case, inflammatory diarrhea was associated with increased tacrolimus exposure (C/D ratio increase ~55%), possibly related to downregulation of intestinal CYP3A4 and P-gp activity. We emphasize that this interpretation remains speculative in the present case. We did not measure intestinal CYP3A4 or P-gp expression, stool inflammatory markers, enteric pathogens, or perform pharmacokinetic absorption studies. When evaluating post-transplant diarrhea, clinicians should not only focus on dehydration but also proactively consider reducing the tacrolimus dose to prevent drug accumulation and toxicity.

### 6.4 Development and application of a structured dose adjustment strategy

When a patient’s clinical condition fluctuates dramatically, multiple physiological and pathological factors may simultaneously influence tacrolimus pharmacokinetics, rendering trough concentration interpretation and dose adjustment particularly complex. In such cases, linear dose adjustment based solely on TDM values is highly prone to failure. The multifactor-based structured dose adjustment strategy proposed in this study (Supplementary Figure S1) integrates assessment of relevant factors and TDM-based validation to form a closed-loop decision-making process. In this complex case, the framework helped organize tacrolimus TDM interpretation across rapidly changing clinical conditions. Its broader clinical value remains to be established in prospective studies.

### 6.5 Limitations

This study has notable limitations. First, it is a single-case observation, and the proposed structured dose-adjustment framework is exploratory and has not been tested in other patients or populations. The findings may not generalize to pediatric recipients, individuals with stable graft function, non-Asian ethnic groups with different CYP alleles, or centers using alternate immunosuppressive protocols. Second, the lack of pharmacokinetic modeling and mechanistic biomarkers prevents definitive causal inferences. Third, it should be noted that the C_std_ provides an estimate of free-drug exposure based on a linear partition model; it does not represent a direct measurement of unbound tacrolimus. The correction formula, has not been prospectively validated in re-transplant recipients with concomitant severe inflammation and fluctuating plasma protein binding. Therefore, corrected values should be interpreted with caution and corroborated with clinical and pharmacodynamic markers.

## 7 Conclusion

Individualized tacrolimus therapy in complex post-liver-transplant cases is a systematic endeavor influenced nonlinearly by multiple factors, including HCT, gastrointestinal function, hepatic metabolic status, genetic polymorphisms, and drug interactions. Our case-based structured framework integrates pharmacological mechanisms and clinical evidence to support tacrolimus TDM interpretation in complex post-liver-transplant scenarios. This framework may help structure clinical reasoning during complex and variable post-transplant courses; however, its impact on clinical outcomes requires prospective evaluation before broader application.

## Data Availability

All relevant data is contained within the article: The original contributions presented in the study are included in the article/Supplementary material, further inquiries can be directed to the corresponding author/s.
