# Supplementary material for: A multifactorial approach to tacrolimus therapeutic drug monitoring in complex liver transplantation: a case report
Source: Front Med (Lausanne). 2026 Jul 24;13:1837572. doi: 10.3389/fmed.2026.1837572 (PMC13448784; doi:10.3389/fmed.2026.1837572)
Supplement: Supplementary file 1 [file Data_Sheet_1.docx]

A Multifactorial Structured Dose Adjustment Strategy for Tacrolimus in Complex Liver Transplantation: Overcoming Challenges in Concentration Interpretation

Limian Liang^1,†^, Tian He^1,†^, Yiming Huang^2,†^, Min Wu^1^, Jiajia Liu^1,^ Shiyun Chen^1^, Wei Li^1^, Miaona Liu^1,*^, Pusen Wang^2,*^

^1^Department of Pharmacy, Shenzhen Third People’s Hospital, Shenzhen, Guangdong Province 518112, China.

^2^Department of Liver Surgery and Organ Transplantation Center, Shenzhen Third People’s Hospital, Shenzhen, Guangdong Province 518112, China.

**†Joint first authorship**

*** Correspondence:**
Miaona Liu
natalie627627@163.com

Pusen Wang

[pusenwang@sjtu.edu.cn](mailto:pusenwang@sjtu.edu.cn)

Keywords: Liver transplantation; Tacrolimus; Therapeutic drug monitoring; Dose adjustment; Complex cases; Pharmacokinetics; hematocrit

**Table S1.** *Recommended Tacrolimus Initial Dosing Strategy Based on CYP3A5 Genotype*

| **CYP3A5 Genotype** | **Metabolic Phenotype** | **Recommended Initial Dose**  **(mg/kg/day, divided Q12h)** | **First TDM Timepoint** |
| --- | --- | --- | --- |
| **1/*1* | Extensive metabolizer (EM) | 0.20–0.30 | POD 3 |
| **1/*3* | Intermediate metabolizer (IM) | 0.15–0.20 | POD 3 |
| **3/*3* | Poor metabolizer (PM) | 0.10–0.15 | POD 5 |

*Abbreviations:CYP3A5, cytochrome P450 3A5; TDM, therapeutic drug monitoring; POD, postoperative day.*

**Figure S1.** Multifactor-based structured tacrolimus dose adjustment framework for complex liver transplant recipients.

*Abbreviations: CYP3A5/CYP3A4, cytochrome P450 3A5/3A4; HCT, hematocrit.DDI, drug–drug interaction; DFI, drug–food interaction;*
